# Possible Tacrolimus-associated seronegative Autoimmune Encephalitis presenting with subacute cognitive impairment and disorientation - case report

**DOI:** 10.3389/fmed.2025.1540960

**Published:** 2025-05-13

**Authors:** Konstantin Hölzl, Francesco Albasini

**Affiliations:** ^1^Medical University of Vienna, Vienna General Hospital, Vienna, Austria; ^2^Epileptology and Neurology Centre, University Hospital of Tübingen, Tübingen, Germany; ^3^Department of Psychiatry and Psychotherapy, University Hospital of Tübingen, Tübingen, Germany; ^4^Department of Public Mental Health, Central Institute of Mental Health, Medical Faculty Mannheim, Heidelberg University, Mannheim, Germany; ^5^Department of Psychiatry and Psychotherapy, Central Institute of Mental Health, Medical Faculty Mannheim, Heidelberg University, Mannheim, Germany; ^6^Central Institute of Mental Health (CIMH), Mannheim, Germany

**Keywords:** Tacrolimus, cognitive impairment, Antibody-negative Autoimmune Encephalitis, altered mental state, liver transplantation

## Abstract

Autoimmune encephalitis (AE) is a multifaceted topic that encompasses various aetiologies and differential diagnoses. In certain instances, the patient’s serum or CSF may not reveal the presence of specific antibodies, which can leave healthcare providers unsure about the most appropriate course of treatment. This report outlines the case of a 71 years-old male who underwent a full-size liver transplantation and was subsequently maintained on Tacrolimus for immunosuppression. The patient presented with symptoms of confusion and decreased general orientation, which worsened over time. Following a thorough diagnostic workup, the patient was diagnosed with Antibody-negative Autoimmune Encephalitis (AE). He received treatment with high-dose steroids and Tacrolimus was later changed to Mycophenolate mofetil (MMF), which ameliorated his condition. This case highlights a potential link between long-term use of calcineurin inhibitors and seronegative AE. Hence, it may be advisable to consider Antibody-negative AE (ABnAE) as a potential diagnosis in patients under chronic immunosuppression who exhibit symptoms of sudden cognitive decline.

## Introduction

### Background

Seronegative Autoimmune Encephalitis (SAE) is defined as an Autoimmune Encephalitis (AE) without detection of specific antibodies (1). It can present a particular challenge to clinicians since its diagnosis is largely relying on the exclusion of other causes. This is particularly challenging in geriatric patients, many of whom have underlying chronic conditions. Moreover, the symptoms of SAE are usually broad and unspecific, encompassing lethargy, altered levels of consciousness, epileptic seizures, and other specific focal neurologic deficits. Additionally, neuropsychiatric symptoms, involuntary movements, memory impairment, and headaches have been reported (2–4). Seronegative AE is categorised into three subgroups: seronegative Limbic Encephalitis (SLE), Antibody-negative probable Autoimmune Encephalitis (AbNAE) and Acute Demyelinating Encephalomyelitis [ADEM; (1, 5)]. In all forms, imaging shows hyperintense lesions in the T2-weighted fluid attenuating inversion recovery sequence (FLAIR) sequence, which, however, differ in their anatomical localization. In AbNAE the lesions should extend only to non-limbic regions of the brain (6).

### Case presentation

The patient, a 71 years-old male, was referred to the gastroenterology department due to symptoms of nausea, vomiting, and diarrhoea over the past 2 weeks. Upon diagnosis, it was revealed that the patient was experiencing a reactivation of a prior cytomegalovirus (CMV) infection, which he likely contracted through a full-size liver transplantation one and a half years earlier. The CMV-infection was successfully treated with high-dose Valganciclovir. It is important to highlight that the patient underwent the transplantation due to NASH liver-cirrhosis complicated by hepatocellular carcinoma (HCC). The underlying cause for the liver cirrhosis was not determined, as all tests for autoimmune and viral hepatitis had come back negative. Since transplantation, the patient had been maintained on Tacrolimus to prevent organ rejection. His medical history also included atrial fibrillation, diastolic heart failure, recurrent urinary tract infections, and acquired dyslipidaemia requiring fibrate therapy. He had no known neurological, psychiatric, or genetic disorders. Prior to his current illness, he was retired and living independently. Upon presentation, no focal neurologic deficits or meningeal signs were noted. The patient arrived with normal vital signs and was alert and oriented to person, location, time, and situation. The patient’s condition deteriorated significantly in the following days, to the point where he became drowsy, disoriented, and complained of severe headaches without any signs of meningeal involvement or other new focal neurological deficits upon an extensive physical examination. As a result, an emergency transfer to the Department of Neurology was necessary. During the stay, the patient experienced focal-clonic seizures in their right arm, followed by one generalized tonic-clonic seizure. Considering the subacute onset and the patient’s immunosuppression, special attention was given to their medical history regarding infections. It was noted that years prior to transplantation, the patient had contracted tuberculosis and toxoplasmosis, both of which had been treated successfully.

### Investigations

**FIGURE 1:**
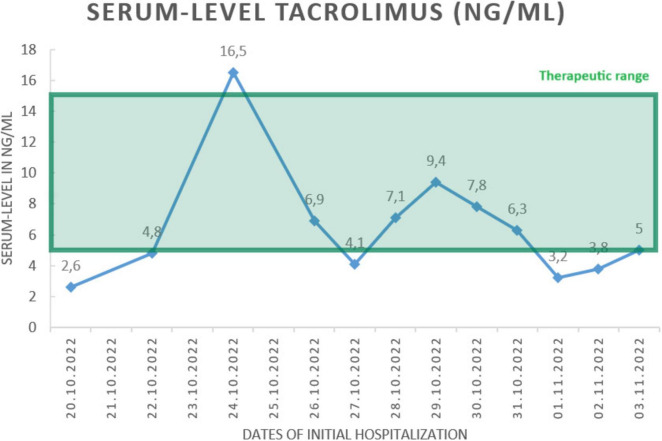
Depiction of Tacrolimus serum-levels (in ng/ml) over the course of the initial hospitalization. As shown above, levels remained mostly within therapeutic range or slightly below normal. In other diseases such as Tacrolimus-associated posterior reversible encephalopathy syndrome (PRES), disease is more likely to occur when higher doses of immunosuppressive medication are routinely administered such as in stem cell transplantation. However, most known cases occur when Tacrolimus serum-levels remain well within the therapeutic range (10, 11). We believe the same is true for Tacrolimus-associated Antibody-negative AE (ABnAE).

Upon admission, laboratory testing was conducted, including a complete blood count, liver and renal function tests, and inflammatory markers. The CRP levels were found to be elevated at 2.35 mg/dl, and thrombocyte count was low at 113,000 per microliter. Additionally, a slight hypocalcaemia was observed at 2.0 mmol/l. Tacrolimus-levels were closely monitored as seen in Figure 1. A native cranial CT was performed at the onset of symptoms, which did not reveal any evidence of intracranial hemorrhage or territorial demarcated ischemia. The cranial MRI, including cranial phlebography, revealed multiple patchy T2 signal enhancements in the subcortical and periventricular white matter on T2-weighted/FLAIR sequence, as well as small disseminated focal enhancements supra- and infratentorial, which were consistent with general inflammatory alterations. The subsequent lumbar puncture revealed clear cerebrospinal fluid with elevated lactate (2.6 mmol/l) and protein levels (225 mg/dl), as well as 13 leucocytes/microliter. The CSF basis parameters suggest that the cause could be either infectious (most likely viral, intracellular bacterial or parasitic) or of inflammatory/autoimmune origin. However, after conducting testing on both serum and CSF, all infectious results came back negative, except for serum-IgG for Borrelia burgdorferi, which was marginally elevated (17.6 RE/ml) and deemed insignificant. Additionally, testing for anti-neuronal antibodies did not yield any results. Neuroimaging showed no indication of elevated CSF-pressure. A brain biopsy was performed in the neurosurgical department, revealing brain tissue with non-specific inflammatory alterations.

During the patient’s stay, shortness of breath was noticed, and a cardiac ultrasound was conducted, which showed chronic heart failure with a reduced EF of less than 35%, first diagnosed approximately 2 years prior. A 24 h ECG was also obtained, revealing atrial fibrillation. As a result, intravenous Heparin was initiated as anticoagulation.

### Differential diagnosis

The primary differential diagnosis consisted of a central nervous system infection caused by both common and opportunistic pathogens. Despite the patient’s history of latent toxoplasmosis and CMV infection, no responsible infectious agent was identified in the serum or CSF. Other potential diagnoses that were considered included antibody-positive autoimmune encephalitis and neurosarcoidosis. Still, the patient received antibiotic and antiviral medication until an infection could be ruled out.

Limbic encephalitis was also considered as a potential diagnosis, as it can be present even without the detection of autoantibodies. Symptoms typically include a subacute onset of altered mental status, short-term memory deficits, confusion, and mood changes. Imaging often reveals bilateral T2/FLAIR-hyperintensities that are confined to the medial aspects of the temporal lobe (5). This was not compatible with the imaging results in our case, which made the diagnosis unlikely.

Sarcoidosis on the other hand is an immune-mediated multisystem disorder and is thought to be triggered by a heightened cell-mediated granulomatous response to a yet unknown antigen. Neurosarcoidosis, which can present as encephalopathy and has a varied appearance on MRI, is usually only seen in combination with other organ manifestations, like in the lungs, skin, or eyes. Furthermore, in the CSF we only found an unspecific pleocytosis, indicating the presence of an inflammation. The analysis of the CD4^+^/CD8^+^ ratio in CSF showed an opposite result to those typical of sarcoidosis with 0,53 and the IL-6 levels were normal (Table 1) (7, 8). The most reliable tool for the diagnosis is a biopsy, which was negative in our patient, who furthermore, presented with no clinical indications for systemic sarcoidosis (9). In particular, there was no evidence of pulmonary sarcoidosis on the chest CT. No skin irritations or ocular disturbances were noted during his stay in our department.

**TABLE 1:** Results of lumbar puncture at initial hospitalization (left) and follow-up visit (right).

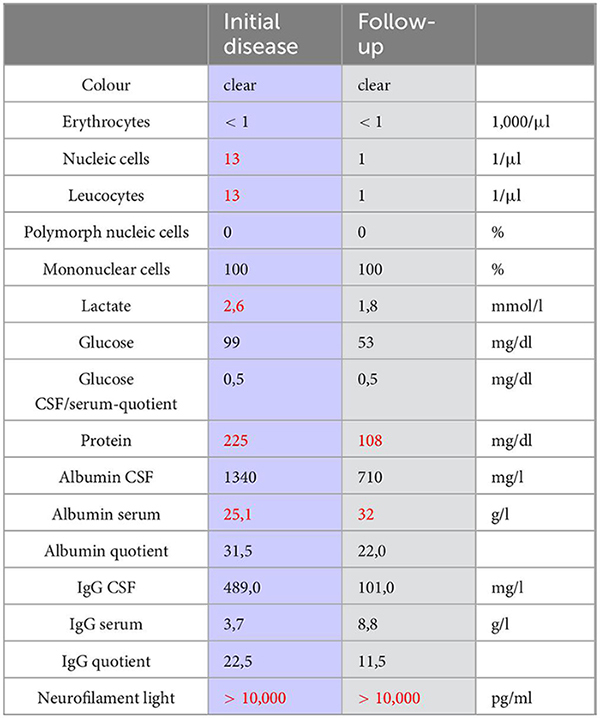

All elevated parameters are marked red.

The possibility of calcineurin inhibitor toxicity leading to posterior reversible encephalopathy syndrome (PRES) was considered given the patient’s non-specific altered mental status, vague headaches, and seizures, despite the absence of visual field deficits. However, literature on PRES following liver transplantation indicates that symptoms generally emerge within the first 2 months post-transplant (10), which contrasts with the clinical timeline in our case. Furthermore, the MRI pattern of oedematous changes in our case was not concentrated in the parietal and occipital lobes, as is usually seen in PRES. Instead, it was more widespread, especially in the subcortical white matter, the periventricular space, as well as multiple small cortical lesions of a contrast-enhancing nature, suggesting an underlying inflammatory disease (11). Moreover, liquor-pleiocytosis as well as the absence of vasogenic oedema on brain biopsy further undermined the likelihood of PRES (12).

Acute disseminated encephalomyelitis (ADEM) was also considered as a possible diagnosis. This is a monophasic demyelinating disease that is more commonly found in children than in adults. In adults, it is common for post-infectious or post-vaccination encephalomyelitis to present with encephalopathy, fever, and meningeal signs. However, it is less common in those over 65 years of age. High IgG levels are often observed. Additionally, the clinical presentation in adults is usually accompanied by long-tract dysfunction, not present in our patient (13).

**FIGURE 2:**
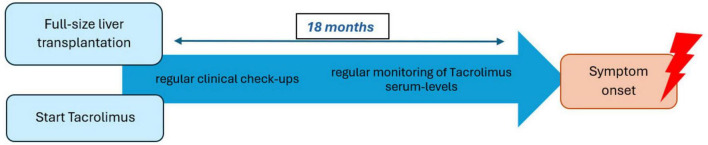
Timeline between full-size liver transplantation to symptom onset.

After the differential diagnostic process, Antibody-negative Autoimmune Encephalitis (AbNAE) was diagnosed using the criteria established by Graus et al. Our patient showed rapid disease progression and presented with altered mental status and neuropsychiatric symptoms. No previously characterized autoantibodies were detected in serum or CSF, even though MRI-findings in combination with the CSF-analysis and the results of the brain biopsy were highly suggestive of autoimmune encephalitis. Additionally, all other reasonable causes were ruled out (5). Upon reviewing the patient’s immunosuppressive regimen, we noted that Tacrolimus was the sole immunomodulatory agent. Given the absence of alternative explanations and the suspected autoimmune mechanism, we decided to replace Tacrolimus with MMF. The Naranjo rating scale (14), a widely used tool for assessing adverse drug reactions (ADR), yielded a score of 6, classifying this as a “probable” adverse reaction to Tacrolimus. The result was determined as follows: +1 point was assigned due to previous reports of similar adverse events, and +2 points were given because the symptoms appeared after drug administration. An additional +1 point was added as symptom amelioration was observed following Tacrolimus discontinuation. Finally, the last +2 points were assigned since all other potential causes were excluded. According to the Liverpool causality assessment tool (15), another scale for detecting ADR, our case was also classified as “probable” using the same metrics as in the Naranjo rating scale. A timeline-model can be viewed in Figure 2.

### Treatment

Immunosuppression with Tacrolimus was discontinued and replaced with MMF. The patient’s liver function and electrolytes were closely monitored. In addition, the patient received two cycles of high-dose steroids (1 gr./day for 3 days and 1 gr./day for 5 days) and was subsequently prescribed oral prednisolone 12.5 mg as an add-on, which will be gradually reduced in the following weeks. Considering the recent onset of seizure activity, the patient has been prescribed Lacosamide 100 mg twice daily.

### Results

Especially after discontinuing immunosuppression with Tacrolimus and administrating the second cycle of high-dose steroids, the patient improved significantly, being more alert and oriented regarding his person again. The patient was transferred in stabilized conditions to a neurological clinic closer to his hometown to later be further transferred to a rehabilitation clinic, with the recommendation of a follow-up visit at our department in 3–4 months. At the time of discharge, no new neurological signs were found.

**FIGURE 3:**
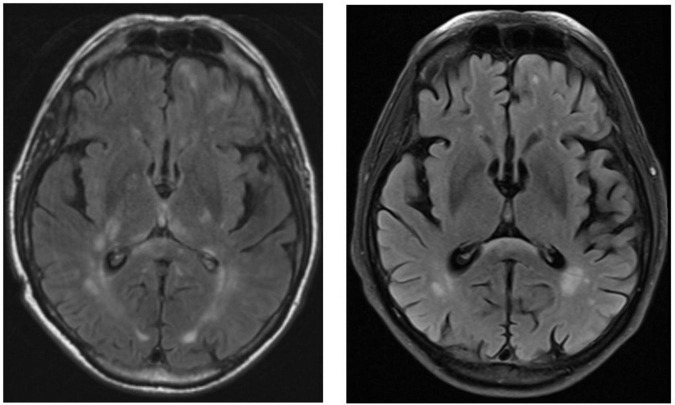
**(Left)** Initial MRI T2 fluid attenuating inversion recovery sequence (FLAIR) image roughly at the level of the basal ganglia showing broad subcortical T2-enhancement, described as of most likely inflammatory aetiology. **(Right)** T2-FLAIR imagine obtained at follow-up visit roughly 4 months later showing strongly regredient T2-enhancement at basal ganglia level.

In the follow up visit after 3 months, the patient was awake, fully oriented and had no apraxia anymore. In the follow up MRI-imaging, the cerebral lesions were regredient, which can be observed in Figure 3. Unfortunately, he had suffered a cardioembolic cerebral stroke, probably by insufficient heparin dose. Besides his newly acquired hemiparesis and new cardioembolic lesions on MRI, no recent signs were found. In the CSF after lumbar puncture, only slightly elevated protein-levels were found (Table 1). Pleocytosis was not present anymore. In a cognitive assessment using the Montreal cognitive Assessment - MoCA - (16), a score of 21/30 points was shown, still indicating a mild cognitive deficit. Altogether, the patient showed great improvement in short- and long-term memory in comparison to his previous hospitalization.

## Materials and methods

We started our research about the general topic of ABnAE after conferring with the patient’s next of kin, informing them of our interest in publishing this case. At the beginning, we summarized the diagnostic work-up we had done and simultaneously looked for similar cases published elsewhere, by searching for “Antibody-negative autoimmune encephalitis” in combination with “liver transplantation” and “immunosuppression/Tacrolimus.” Throughout the project we paid close attention to existing guidelines for writing single case publications by Gagnier et al. This case report might be limited by the complexity of the case presented, first regarding the patient’s vast medical history and plethora of pre-existing conditions and secondly by the intricate course of disease progression exhibited during the stay in the hospital. Still, it remains an interesting description of a highly unusual combination of symptoms and clinical findings, possibly pointing toward a mechanism of injury that is so far not widely considered.

## Discussion

In our investigation, we found only one case report for AbNAE related to chronic immunosuppression after transplantation. Spindel et al. presented a case of a patient with confusion, lethargy, light-headedness, and memory deficits while being on Tacrolimus for over a decade after receiving a liver transplant some 14 years prior. After receiving high-dose intravenous methylprednisolone for 5 days the patients mental status began to improve, and he went on to recover completely (4).

We found three other case reports concerning AbNAE. One was describing a 63 years-old male with no underlying conditions, presenting with disorientation in time and space, as well as memory deficits, headaches, and nausea. Although all tests for pathogens or autoantibodies were negative in CSF and serum, MRI showed bilateral T2-hyperintensities in the temporal cortex, the putamen, the hippocampi, the medial thalami, with neither diffusion deficit nor contrast enhancement. Even after suspecting AbNAE and accordingly treating the patient with high-dose steroids, the patient’s condition kept deteriorating to a comatose state combined with generalized tonic-clonic seizures. After a 2 weeks attempt with intravenous Immunoglobulin (IVIGs) failed with the patient showing no improvement, treatment with Rituximab was initiated. After 3 weeks the patient’s condition started to improve and eventually got to a point where he could be transferred to a rehabilitation facility. Blood B-cell counts (CD19^+^/CD20^+^) were measured before Rituximab administration and amounted to 15.3% and 15.1% of all lymphocytes, respectively. Five months later, the alterations on MRI appeared substantially regressed, and the B-cell count (CD19^+^/CD20^+^) remained at 0%, indicating a sufficient B cell-depleting effect of Rituximab (3).

The other two reports involved considerably younger patients. One being a 35 years-old, who presented with two different episodes of behavioral and speech disturbance and mental confusion over a period of 6 months, which were treated with high-dose methylprednisolone and IVIGs. Subsequently, some symptoms ameliorated slightly even though the patient’s cognitive abilities showed no satisfying recovery, as the patient scored five points in the MoCA at his last follow-up (17). Another patient, a 20 years-old female, presented with subacute onset of psychiatric symptoms, such as anxiety, in combination with mental confusion, memory deficits and seizures. After establishing the diagnosis, treatment with high-dose methylprednisolone was started, followed by seven sessions of plasma exchange, after which her condition improved significantly, and she was discharged on 15 mg of oral prednisolone (2).

In summary, it seems that the cases presented contain some similarities, but no clear pattern in terms of effective therapy or diagnostic pathway was observable within the literature. The case described by Spindel et al. shares several clinical similarities with our patient, particularly in terms of disease onset, symptomatology, and response to treatment. This parallel led us to further investigate the potential link between long-term Tacrolimus therapy and the development of autoimmune encephalitis, including seronegative forms. To explore this hypothesis, we reviewed additional cases of AbNAE reported in the literature. The authors hypothesized the long-term immunosuppression, mainly focused on suppressing T-cell function, to have created an imbalance between T- and B-cells and thereby increasing the likelihood of developing B-cells without immune tolerance. Literature on Antibody positive AE has concluded that complement-independent antibody effects are most likely responsible for its development. If a similar mechanism applies to AbNAE, an imbalance favoring B-cell activation could contribute to disease progression or even predispose patients to its development (4, 18, 19). Additionally, it was reported that Rituximab, which is a monoclonal antibody against CD20^+^-cells, was successfully used as treatment in AbNAE (3).

Previous studies suggest that in patients treated with calcineurin inhibitors after a living-donor liver transplant (LDLT), the number of regulatory T-cells decreased over the course of the first weeks after the start of the medication, whereas all other lymphocytic cell lines remained leveled [(20, 21)]. Calcineurin is a protein phosphatase and possesses several important roles in regulation of the immune system, one of which is the activation of the IL-2 pathway. By Inhibition of Calcineurin production of IL-2 is suppressed, which is crucial for the induction of regulatory T-cells. In contrast to that, other cell lines such as TH17^+^-cells seemed to be almost unaffected by immunosuppression leading to increased production of effector memory B-cells through IL-17. Consequently, Calcineurin inhibitors-induced suppression of T-cell function may result in an imbalance that favors B-cell activation, potentially facilitating the development of antibody-negative autoimmune encephalitis (21–23).

After diagnosing AbNAE, prediction of the disease course and the overall prognosis is difficult and often unreliable. Using EEG as a monitoring tool for disease progress, seems to work in one instance, but might not be very reliable and practical in a broad sense (2). Apparently, B-cell counts might also be connected to disease intensity and could serve as a valuable diagnostic tool. Whether it contains significant predictive value is yet to be determined. Furthermore, studies of patients suffering from Antibody positive AE suggests that so-called neutrophil-to-lymphocyte ratio (NLR) and monocyte-to-lymphocyte ratio (MLR) taken from peripheral blood are both positively correlated with disease intensity. This could be another approach to help us better understanding the progression and outcome of the disease in the future (24).

In conclusion, diagnosis of SAE is difficult even for experienced clinicians, since a plethora of non-specific and broad symptoms are usually present, and no clear diagnostic marker is available to distinguish it from more common differential diagnoses.

We suspect that through the possible mechanism of injury postulated earlier, long-term immunosuppression with Calcineurin inhibitors could trigger an immune response resulting in the clinical scenario we just described, even if the therapeutic window is strictly adhered to. Given the diagnostic challenges associated with AbNAE and the lack of specific biomarkers, further studies are essential to establish standardized diagnostic criteria and effective treatment strategies. Although current understanding is largely based on isolated case reports, prospective studies investigating the long-term effects of calcineurin inhibitors on immune function could provide valuable insights into the underlying pathophysiology. Until then, finding the correct diagnosis and successfully treating it will depend heavily on clinicians being able to rely on the knowledge gathered from isolated case reports published in the literature.

## Learning points

1. AbNAE should be seen as an important differential diagnosis especially in patients with altered mental state.
2. Immune system dysregulation caused by immunosuppressive medication might be directly responsible for some cases of ABnAE.
3. Elevated serum-levels of Tacrolimus may increase the likelihood of illness, but this condition may also occur when serum-levels are within the therapeutic range.
4. In such cases, discontinuation or change in immunosuppressive drugs should be discussed.

## Data Availability

The raw data supporting the conclusions of this article will be made available by the authors, without undue reservation.

## References

[1] Lee WJ Lee HS Kim DY Lee HS Moon J Park KI Seronegative autoimmune encephalitis: clinical characteristics and factors associated with outcomes. *Brain.* (2022) 145:3509–21. 10.1093/brain/awac166 35512357

[2] Mito M Sakurai K Nakamura Y Nagai A Seo S Tanaka K EEG contribution to the diagnosis of antibody-negative autoimmune encephalitis: a case report. *Case Rep Neurol.* (2021) 13:739–43. 10.1159/000519991 35082642 PMC8739390

[3] Park SH Kim YC. Case report: the use of rituximab in antibody-negative autoimmune encephalitis. *Front Neurol.* (2021) 12:686009. 10.3389/fneur.2021.686009 34539548 PMC8442530

[4] Spindel J Heckroth M Marsano L. Antibody-negative autoimmune encephalitis as a complication of long-term immune-suppression for liver transplantation. *BMJ Case Rep.* (2020) 13:e235777. 10.1136/bcr-2020-235777 32933909 PMC7493118

[5] Graus F Titulaer MJ Balu R Benseler S Bien CG Cellucci T A clinical approach to diagnosis of autoimmune encephalitis. *Lancet Neurol.* (2016) 15:391–404. 10.1016/S1474-4422(15)00401-9 26906964 PMC5066574

[6] van Steenhoven RW Titulaer MJ. Seronegative autoimmune encephalitis: exploring the unknown. *Brain.* (2022) 145:3339–40. 10.1093/brain/awac338 36111366 PMC9586533

[7] Chazal T Costopoulos M Maillart E Fleury C Psimaras D Legendre P The cerebrospinal fluid CD4/CD8 ratio and interleukin-6 and -10 levels in neurosarcoidosis: a multicenter, pragmatic, comparative study. *Eur J Neurol.* (2019) 26:1274–80. 10.1111/ene.13975 31021023

[8] Nordström S Andersson B Malmeström C. Cerebrospinal fluid CD4+ /CD8+ ratio in diagnosing neurosarcoidosis. *Acta Neurol Scand.* (2020) 142:480–5. 10.1111/ane.13297 32533774

[9] Giovannoni G Chamoun V Scadding J Thompson E. Neurosarcoidosis. In: editor s *Sarcoidosis*. Boca Raton, FL: Taylor & Francis group (2012). p. 256–70.

[10] Wu Q Marescaux C Wolff V Jeung MY Kessler R Lauer V Tacrolimus-associated posterior reversible encephalopathy syndrome after solid organ transplantation. *Eur Neurol.* (2010) 64:169–77. 10.1159/000319032 20699617

[11] Fischer M Schmutzhard E. Posterior reversible encephalopathy syndrome. *J Neurol.* (2017) 264:1608–16. 10.1007/s00415-016-8377-8 28054130 PMC5533845

[12] Datar S Singh TD Fugate JE Mandrekar J Rabinstein AA Hocker S. Albuminocytologic dissociation in posterior reversible encephalopathy syndrome. *Mayo Clinic Proc.* (2015) 90:1366–71. 10.1016/j.mayocp.2015.07.018 26349950

[13] Berzero G Cortese A Ravaglia S Marchioni E. Diagnosis and therapy of acute disseminated encephalomyelitis and its variants. *Expert Rev Neurother.* (2016) 16:83–101. 10.1586/14737175.2015.1126510 26620160

[14] Naranjo CA Busto U Sellers EM Sandor P Ruiz I Roberts EA A method for estimating the probability of adverse drug reactions. *Clin Pharmacol Ther.* (1981) 30:239–45. 10.1038/clpt.1981.154 7249508

[15] Um SH Abuzgaia A Rieder M. Comparison of the Liverpool Causality Assessment Tool vs. the Naranjo Scale for predicting the likelihood of an adverse drug reaction: a retrospective cohort study. *Br J Clin Pharmacol.* (2023) 89:2407–12. 10.1111/bcp.15704 36849649

[16] Freitas S Simões MR Marôco J Alves L Santana I. Construct validity of the Montreal Cognitive Assessment (MoCA). *J Int Neuropsychol Soc.* (2012) 18:242–50. 10.1017/S1355617711001573 22115097

[17] Sequeira C Lopes P. Antibody negative autoimmune encephalitis: a case report. *Acta Med Portuguesa.* (2021) 33:A1–5. 10.20344/AMP.13793 33382365

[18] Konen FF Schwenkenbecher P Jendretzky KF Hümmert MW Wegner F Stangel M Severe anti-N-methyl-D-aspartate receptor encephalitis under immunosuppression after liver transplantation. *Front Neurol.* (2019) 10:987. 10.3389/fneur.2019.00987 31608003 PMC6773799

[19] Saiz A Graus F. Neurologic complications of hematopoietic cell transplantation. *Semin Neurol.* (2010) 30:287–95. 10.1055/s-0030-1255218 20577935

[20] Han JW Joo DJ Kim JH Rha MS Koh JY Park HJ Early reduction of regulatory T cells is associated with acute rejection in liver transplantation under tacrolimus-based immunosuppression with basiliximab induction. *Am J Transpl.* (2020) 20:2058–69. 10.1111/ajt.15789 31965710

[21] Kim HY Cho M Jhun JY Byun JK Kim EK Yim YB The imbalance of T helper 17/regulatory T cells and memory B cells during the early post-transplantation period in peripheral blood of living donor liver transplantation recipients under calcineurin inhibitor-based immunosuppression. *Immunology.* (2013) 138:124–33. 10.1111/imm.12021 23205589 PMC3575765

[22] Kincaid RL. *Special lectures the role of calcineurin in immune system responses*. Potomac, MD: Veritas, Inc (1995). 10.1016/s0091-6749(95)70202-4 8543774

[23] Wallin EF Hill DL Linterman MA Wood KJ. The calcineurin inhibitor tacrolimus specifically suppresses human T follicular helper cells. *Front Immunol.* (2018) 9:1184. 10.3389/fimmu.2018.01184 29904381 PMC5990622

[24] Liu Z Li Y Wang Y Zhang H Lian Y Cheng X. The neutrophil-to-lymphocyte and monocyte-to-lymphocyte ratios are independently associated with the severity of autoimmune encephalitis. *Front Immunol.* (2022) 13:911779. 10.3389/fimmu.2022.911779 35844590 PMC9283563

